# ^1^H-NMR-Based Metabolomic Study for Identifying Serum Profiles Associated with the Response to Etanercept in Patients with Rheumatoid Arthritis

**DOI:** 10.1371/journal.pone.0138537

**Published:** 2015-11-11

**Authors:** Roberta Priori, Luca Casadei, Mariacristina Valerio, Rossana Scrivo, Guido Valesini, Cesare Manetti

**Affiliations:** 1 Department of Internal Medicine and Medical Specialties—Rheumatology Unit, Sapienza University of Rome, Rome, Italy; 2 Department of Chemistry—Sapienza University of Rome, Rome, Italy; The Francis Crick Institute, UNITED KINGDOM

## Abstract

**Objective:**

A considerable proportion of patients with rheumatoid arthritis (RA) do not have a satisfactory response to biological therapies. We investigated the use of metabolomics approach to identify biomarkers able to anticipate the response to biologics in RA patients.

**Methods:**

Due to gender differences in metabolomic profiling, the analysis was restricted to female patients starting etanercept as the first biological treatment and having a minimum of six months’ follow-up. Each patient was evaluated by the same rheumatologist before and after six months of treatment. At this time, the clinical response (good, moderate, none) was determined according to the EUropean League Against Rheumatism (EULAR) criteria, based on both erythrocyte sedimentation rate (EULAR-ESR) and C-reactive protein (EULAR-CRP). Sera collected prior and after six months of etanercept were analyzed by ^1^H-nuclear magnetic resonance (NMR) spectroscopy in combination with multivariate data analysis.

**Results:**

Twenty-seven patients were enrolled: 18 had a good/moderate response and 9 were non responders according to both EULAR-ESR and EULAR-CRP after six months of etanercept. Metabolomic analysis at baseline was able to discriminate good, moderate, and non-responders with a very good predictivity (Q^2^ = 0.68) and an excellent sensitivity, specificity, and accuracy (100%). In good responders, we found an increase in isoleucine, leucine, valine, alanine, glutamine, tyrosine, and glucose levels and a decrease in 3-hydroxybutyrate levels after six months of treatment with etanercept with respect to baseline.

**Conclusion:**

Our study confirms the potential of metabolomic analysis to predict the response to biological agents. Changes in metabolic profiles during treatment may help elucidate their mechanism of action.

## Introduction

Over the last years, evidence has increased that early recognition and management of patients with rheumatoid arthritis (RA) leads to better clinical and radiographic outcomes [1–7]. Starting with COBRA trial [3,5,6], the concept of “window of opportunity” has been well recognized, in which rapid suppression of disease decreases or resets the rate of joint damage for years to come [8]. However, many patients fail to adequately respond to treatment [9,10], and reliable biomarkers that accurately predict the response to therapy in individual patients are necessary to help in decision-making [11]. Indeed, with the introduction of a wide spectrum of new, generally expensive drugs, the era of “personalized medicine” for RA patients has become an urgent necessity [12].

In this regards, the analysis of metabolomic profiling may be a tool of utmost value. Metabolomics is based on the evaluation of biological fluids by analytical methods that allow describing a patient's metabolic profile without first having to identify markers of the disease [13]. Mass spectrometry (MS) and nuclear magnetic resonance (NMR) techniques, currently used to finalize the metabolomic analysis, provide both the analytical profiles that reveal the amount of each metabolite, and the correlations among metabolites through the multivariate statistical analysis of spectroscopic signals [14]. The descriptors identified along these lines become the coordinates of a new system of reference represented by metabolomic maps on which patients and their response to therapy are located.

Metabolomics has already been applied to several disorders, including autoimmune diseases and osteoarthritis among rheumatological conditions [15,16,17]. In this setting, metabolomic analysis in distinct biological fluids showed the potential to discriminate patients with different disease activity or different diseases, and to predict the prognosis or the response to treatments [18–27]. In relation to this last point, some studies show that metabolic changes may predict the efficacy of both traditional DMARDs, such as methotrexate (MTX) [24], and biological agents [26] in patients with RA. At present, the Kapoor’s study [26] remains the only one evaluating the potential usefulness of metabolomics in patients treated with biological agents.

Given the ever-expanding use of these therapies, for whom approximately 30–40% of patients subsequently develop an inadequate response [28–33], we decided to enrich the current knowledge with this study. Our aim was to assess whether a ^1^H-NMR-based metabolomic analysis in serum from patients with RA could predict the response to the anti-TNF fusion protein etanercept evaluated at six months.

## Patients and Methods

### Patients

Adult patients with a diagnosis of RA according to the 1987 revised classification criteria of the American College of Rheumatology [34] and designated to start anti-TNF therapy were prospectively enrolled from the rheumatology outpatient clinic at Sapienza University of Rome, Italy. Due to gender differences in metabolomic profiles [35,36], the present analysis was restricted to female patients with active disease starting etanercept as the first biological treatment and having a minimum of six months’ follow-up. Patients were given etanercept by 50 mg subcutaneous doses once weekly while continuing to assume anti-rheumatic medications (DMARDs and/or oral glucocorticoids) as required per the clinical judgment of the treating physician. Each patient was evaluated by the same rheumatologist at baseline before starting etanercept and after six months from the onset of biological treatment. At recruitment data on demographics, diet regimen, disease duration, co-morbidities, and concomitant treatments were obtained by direct questioning and collected on a standardized electronic form. Clinical evaluation in RA patients included: swollen and tender joint count (0–28), patient and physician global assessment on a visual analogue scale (VAS, 0–100 mm), and Health Assessment Questionnaire disability index (HAQ) [37]. Data including anti-citrullinated protein/peptide antibodies (ACPA) and rheumatoid factor (RF) were obtained from the medical records. Each patient underwent a blood drawing to appraise erythrocyte sedimentation rate (ESR) and C-reactive protein (CRP); furthermore, sera samples were obtained and immediately stored at -80°C until the metabolomic analysis was performed. Disease activity score (28 joint count, four variables; DAS28) was calculated and the clinical response (good, moderate, none) was evaluated according to the EUropean League Against Rheumatism (EULAR) criteria [38]. After six months of etanercept therapy, RA patients were divided into two groups according to the clinical response: we merged good and moderate categories as response against no response.

The study received Policlinico Umberto I Ethics Committee approval in accordance with local requirements (prot. n. 589/14) and written informed consent was obtained from each patient.

### Metabolomic analysis

The serum samples were immediately frozen after collection and stored at -80°C. Serum samples were thawed at room temperature and 450 μL of each was added to 400 μL of 0.90% w/v NaCl and of 20% v/v D_2_O (99.9 atom % of deuterium) solution. Each sample was stirred and then centrifuged at 13000g for 10 minutes. Finally, 600 μL of the supernatant was transferred into 5 mm NMR tube for the analysis.

2D ^1^H J-resolved (JRES) NMR spectra were acquired on a 500 MHz VNMRS Varian/Agilent spectrometer (Agilent, Santa Clara, CA) at 25°C using a double spin echo sequence with pre-saturation for water suppression and 16 transients per increment for a total of 32 increments. These were collected into 16 k data points using spectral widths of 8 kHz in F2 and 64 Hz in F1. Each free induction decay (FID) was Fourier transformed after a multiplication with sine-bell window functions in both dimensions. JRES spectra were tilted by 45°, symmetrised about F1, referenced to lactic acid at δH = 1.33 ppm and the proton-decoupled skyline projections (p-JRES) exported using Agilent VNMRJ 3.2 software. The exported p-JRES were aligned, corrected for baseline offset and then reduced into spectral bins with widths ranging from 0.02 to 0.06 ppm by using the ACD intelligent bucketing method (1D NMR Manager software, ACD/Labs, Toronto, Canada). This method sets the bucket divisions at local minima (within the spectra) to ensure that each resonance is in the same bin throughout all spectra. The area within each spectral bin was integrated and, to compare the spectra, the integrals derived from the bucketing procedure were normalized to the total integral region, following exclusion of bins representing the residual water peak δ (4.33–5.17 ppm).

### Statistical Analysis

The resulting data was used as input for univariate and multivariate analysis, including *t*-test and analysis of variance (ANOVA), principal component analysis (PCA), and orthogonal projections to latent structures discriminant analysis (OPLS-DA). PCA and OPLS-DA were performed using SIMCA-P + v.13.0.3 (Umetrics, Umeå, Sweden), SYSTAT v.13 software (Systat Software Inc.) was used for the ANOVA test. Clinical data are expressed as median/range; comparison between responders and non-responders was analyzed with the Mann-Whitney test. A value of p<0.05 was considered to indicate a statistically significant difference.

PCA is a projection method used for exploiting the information embedded in multidimensional data sets [39]. The data is reduced to a few latent variables (or principal components, PCs) collecting the information implicit in the original variables correlation structure. The presence of correlations between the original variables allows for the reduction of dimensionality of the data set in the new space without noticeable loss of information. The extracted PCs are each orthogonal and ordered in terms of percentage of explained variation, with the first components collecting the ‘signal’ (correlated) portion of information, while minor components can be considered as ‘noise’ components. Because PCs are, by construction, orthogonal to each other, a clear-cut separation of the different and independent features characterizing the data set is made possible. Each statistical unit is assigned a score relative to each extracted component. The output from the PCA analysis consists of score plots, which provide an indication of the differences between the classes in terms of metabolic similarity.

OPLS-DA is a supervised pattern recognition technique, widely used in the field of metabolomics to interpret large multivariate data sets describing differences between the groups under study in a straightforward and accurate way. OPLS-DA separates the systematic variation in the matrix X (spectroscopic data) into two parts, one linearly related (variation of interest) to the matrix Y (the classification variables) and one orthogonally related (so-called orthogonal variation or structured noise) to the matrix Y. The partitioning of the X-data improves the interpretation of the model. For each OPLS-DA models, the variances related to the matrix Y are explained by latent variables (LVs).

The influence of the original variables on the obtained model was determined by using variable importance in the projection (VIP) values (VIP>1).

Metabolites responsible for the separation between classes were identified using an in-house NMR database and Chenomx NMR suite v. 7.7 (Chenomx Inc., Alberta, Canada).

## Results

The baseline demographics and disease characteristics of the patients (n = 27) are represented in Table 1. Female patients only were included (median age 60.5 years, range 32–78).

According to EULAR-ESR or EULAR-CRP response criteria, we classified RA patients treated with etanercept as responders (including good and moderate) and non-responders after 6 months of treatment. The two criteria were consistent in dividing patients into 18 responders and 9 non-responders (Table 1), with some differences in the classification of good and moderate responders.

**Table 1:** Baseline characteristics of rheumatoid arthritis patients (n = 27) by response to etanercept therapy at six months.

	Good/moderate response* to etanercept (n = 18)	No response* to etanercept (n = 9)
**Age (yrs; median/range)**	60.5/32-78	62/43-70
**Disease duration** (months; median/range)	72/16-288	18/54-228
**RF positive** (n/%)	12/66.7	7/77.8
**ACPA positive** (n/%)	11/61.1	6/66.7
**DAS28-ESR** (median/range)	5.19/1.26–6.41	5.3 (4.9–7.36)
**DAS28-CRP** (median/range)	4.56/2.27–5.73	4.65/4.36–6.58
**HAQ** (median/range)	0.93/0-2.6	1/0.13–1.38
**Patient global assessment** (VAS, 0–100 mm) (median/range)	60/17-100	60/26-72
**Physician globl assessment** (VAS, 0–100 mm) (median/range)	41.5/18-70	47/35-78
**Concomitant treatment regimen** (n/%)		
Glucocorticoids	4/22.2	1/11.1
DMARDs	6/33.3	0
DMARDs and glucococorticoids	7/38.9	6/66.7
No immunosuppressants	1/5.5	2/22.2
**Daily glucocorticoid dose** ** (mg; median/range)	5/2-25	5/5-25
**Weekly methotrexate dose** (mg; median/range)	15/7.5–15	12.5/10-15
**Mediterranean diet** (n/%)	18/100	9/100
**Ever smokers** (n/%)	6/33.3	2/22.2

*As determined by EUropean League Against Rheumatism criteria.

**Prednisone equivalent.

Ever smokers include past and current smokers.

There were no significant differences between the two groups (comparison assessed with Mann-Whitney test for independent samples).

RF: rheumatoid factor; ACPA: anti-citrullinated protein/peptide antibodies; DAS28: disease activity score based on 28 joint counts; CRP: C-reactive protein; ESR: erythrocyte sedimentation rate; DMARDs: disease-modifying anti-rheumatic drugs [include for responders and non responders, respectively: methotrexate (10 and 4), sulfasalazine (1 and 0), hydroxychloroquine (3 and 0), cyclosporine (1 each), leflunomide (0 and 1]; HAQ: Health Assessment Questionnaire; VAS: visual analogue scale.

### Prediction of Response to Etanercept Therapy

**Fig 1:**
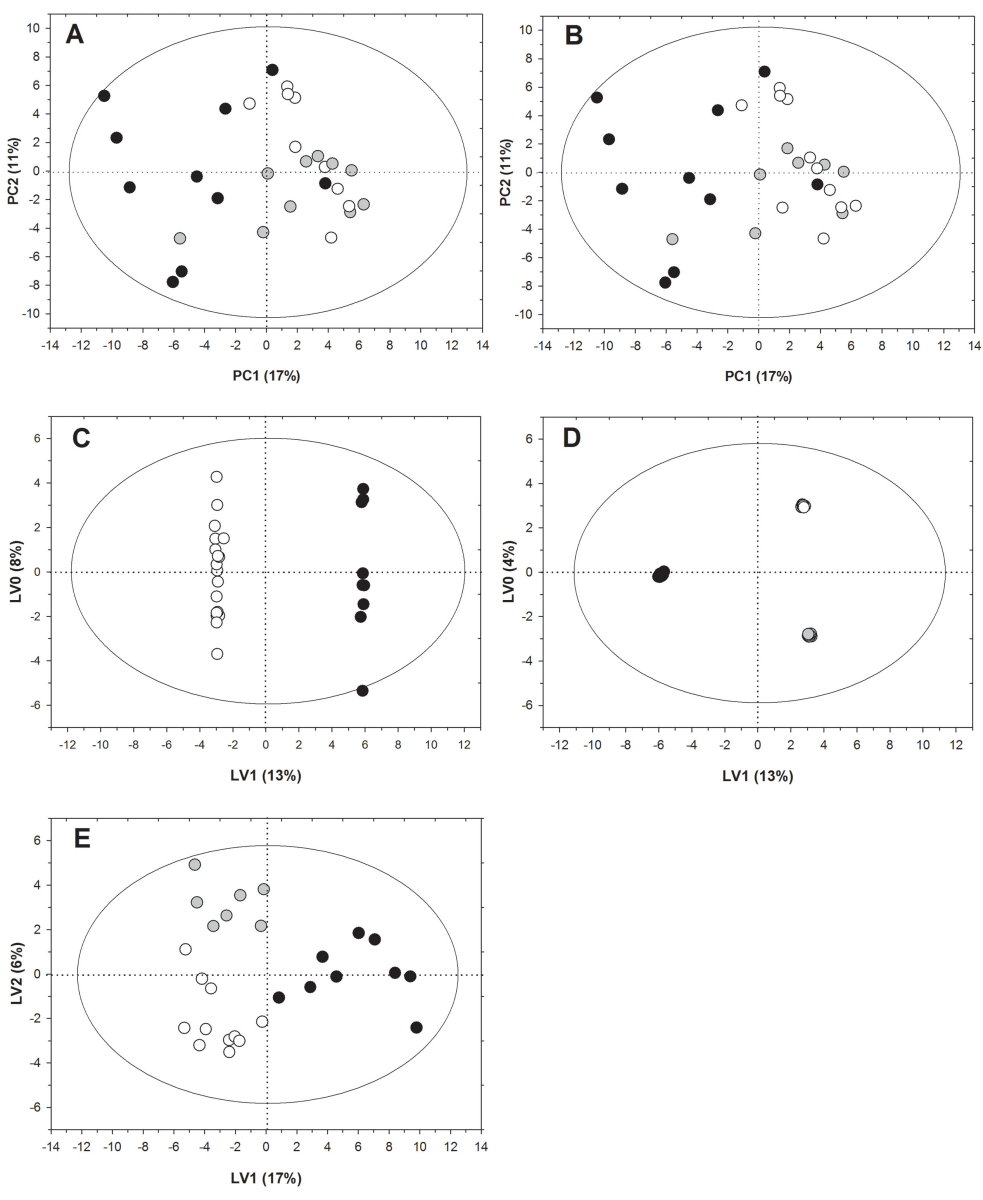
^1^H-NMR metabolomic fingerprinting of RA patients before treatment with etanercept. (A) score plot from PCA analysis according to EULAR-ESR; (B) score plot from PCA analysis according to EULAR-CRP; for A and B white circles represent good responders, gray circles represent moderate responders, black circles represent non-responders. (C) score plot from OPLS-DA separating responder and non-responder patients according to both EULAR-ESR and EULAR-CRP criteria. White circles represent responders (good and moderate) and black circles represent non-responders. (D) score plot from OPLS-DA separating good, moderate, and non-responders according to EULAR-ESR; (E) score plot from OPLS-DA separating good, moderate, and non-responders according to EULAR-CRP criteria, respectively; for D and E white circles represent good responders, gray circles represent moderate responders, black circles represent non-responders.

To identify metabolomic signatures that could predict the response of RA patients to etanercept, we applied PCA to NMR spectra of serum samples collected at baseline. PCA is an unsupervised method useful to investigate interrelationship among groups through the detection of potential clusters and outliers. By using this method, we obtained a model explaining 42% of the solution variance with four PCs. We then applied a *t*-test to the component scores in order to compare all the specific pairs of patient groups. According to both EULAR-ESR and EULAR-CRP criteria, the results highlighted significant differences between patients who did or did not respond to etanercept on the first PC (PC1) (p<0.0001). However, PCA did not provide discrimination between moderate and good responders. Fig 1A and 1B show the score plots from PCA analysis for EULAR-ESR and EULAR-CRP response criteria, respectively.

Since several subject-related factors such as age, smoking, and dietary habits as well as concomitant treatments and co-morbidities are known to affect the metabolome [40], we decided to assess whether any of these could explain the separation between RA patients responding and non-responding to etanercept. To this purpose, the metabolic profiles and the above-mentioned factors were compared for each patient by ANOVA test (Table 2). This analysis revealed that only co-morbidity, found in 90% of non-responders against 59% of responding patients, affected the clustering of metabolic profiles (p = 0.041). However, by using Pearson’s simple and partial correlation analysis to simultaneously investigate PCA score, responder or non-responder status, and co-morbidities, we inferred that the metabolic variations on PC1 were mainly associated with the prediction of response to etanercept. Indeed, after adjusting for co-morbidities, the correlation coefficient between PCA score and the clinical status dropped from 0.63 to 0.60, thus emphasizing the concept that the main driver of metabolism-based classification was represented by the condition of being responder or non-responder to biological treatment.

**Table 2:** Variability of the metabolomic profiles in terms of potential confounding variables in the four principal components identified by PCA and expressed as F-values together with the corresponding p-values (in brackets).

	PRINCIPAL COMPONENTS			
	PC1	PC2	PC3	PC4
Age	0.296 (0.591)	0.084 (0.775)	0.102 (0.753)	1.115 (0.301)
Smoking	0.214 (0.808)	0.999 (0.383)	0.424 (0.659)	0.714 (0.500)
Dietary habits	0.969 (0.471)	0.190 (0.976)	1.792 (0.152)	0.919 (0.502)
Concomitant treatment	0.776 (0.552)	0.464 (0.762)	0.579 (0.681)	1.226 (0.329)
Co-morbidity	4.635 (**0.041**)	0.940 (0.341)	0.270 (0.608)	0.184 (0.672)
ACPA	2.136 (0.156)	0.016 (0.899)	0.173 (0.681)	0.417 (0.524)
RF	4.287 (**0.049**)	0.013 (0.909)	0.004 (0.952)	0.085 (0.773)
Disease duration	0.033 (0.857)	2.409 (0.133)	1.400 (0.248)	0.657 (0.425)

ANOVA test, significant values (p<0.05) are in bold.

Smoking, co-morbidity, ACPA and RF variables are defined as presence or absence; dietary habits variable is defined as mediterranean diet or non-mediterranean diet; age and disease duration variables are defined as numerical values (number of months); concomitant treatment variable is defined as four groups: glucocorticoids, DMARDs, DMARDs and glucocorticoids and no immunosuppressants.

Among subject-related and disease-related factors, co-morbidity and RF, respectively, affected the clustering of metabolic profiles in terms of a minor likelihood of response to treatment in patients positive for either of them. However, by using Pearson’s simple and partial correlation analysis to simultaneously investigate PCA score, responder or non-responder status, and co-morbidities, the metabolic variations on PC1 were mainly associated with the prediction of response to etanercept.

ACPA: anti-citrullinated protein/peptide antibodies; RF: rheumatoid factor.

It is also known that some disease-related factors (ACPA and RF status, disease duration) may influence the treatment response in patients with RA [41–44]. By using ANOVA test, we observed that only RF could affect the response to etanercept, with RF-positive patients being less likely to respond to treatment (Table 2). Furthermore, to analyze the association of metabolic profiles with inflammation, we performed a correlation between the four PCA scores and the parameters incorporated into the EULAR-CRP and EULAR-ESR responses by using ANOVA test (Table 3). The results showed a correlation between PC3 scores and CRP levels (F = 4.812; p = 0.038) and between PC1 scores and tender joint count (F = 7.663; p = 0.011). This last parameter only could affect the response to etanercept: in fact, the median tender joint count 28 (range) was 8 (0–21) and 12 (6–25) for responder and non-responder groups, respectively.

**Table 3:** Variability of metabolomic profiles in terms of variables related to clinical and laboratory parameters in the four principal components identified and expressed as F-values together with the corresponding p-values (in brackets).

	PRINCIPAL COMPONENTS			
	PC1	PC2	PC3	PC4
ESR	0.133 (0.718)	0.087 (0.771)	0.993 (0.329)	0.332 (0.570)
CRP	0.709 (0.408)	0.131 (0.721)	**4.812 (0.038)**	0.000 (0.989)
Tender joint count	**7.663 (0.011)**	1.803 (0.191)	1.406 (0.247)	0.173 (0.681)
Swollen joint count	0.739 (0.398)	2.363 (0.137)	0.102 (0.753)	0.008 (0.928)
Doctor’s global VAS	0.106 (0.747)	1.637 (0.212)	0.325 (0.574)	0.215 (0.647)
Patient’s global VAS	0.519 (0.478)	0.001 (0.970)	0.000 (0.988)	1.278 (0.269)
HAQ	1.165 (0.291)	2.773 (0.108)	1.380 (0.251)	0.384 (0.541)
DAS28-ESR	1.565 (0.222)	0.330 (0.571)	0.362 (0.553)	0.015 (0.903)
DAS28-CRP	1.613 (0.216)	1.637 (0.212)	1.144 (0.295)	0.049 (0.827)

ANOVA test, significant values (p<0.05) are in bold.

The results showed a correlation between PC3 scores and CRP levels and between PC1 scores and tender joint count. This last parameter only could affect the response to etanercept: in fact, the median tender joint count 28 (range) was 8 (0–21) and 12 (6–25) for responder and non-responder groups, respectively.

ESR: erythrocyte sedimentation rate; CRP: C-reactive protein; VAS: visual analogue scale; HAQ: Health Assessment Questionnaire; DAS28: disease activity score based on 28 joint counts.

After having observed a good natural clustering of the metabolic profiles with PCA, we decided to analyze the data by using a supervised model, OPLS-DA. When considering two classes of patients, i. e. responders (good and moderate) and non-responders, we obtained a robust predictive model (Q^2^ = 0.82) with 1 predictive and 5 orthogonal LVs explaining 45% and 32% of the total variability, respectively. The prognostic performance of the response to therapy evaluated by leave-one-out analysis showed 100% sensitivity and specificity (S1 Table). The score plot in Fig 1C shows a clear separation between responder and non-responder patients according to both EULAR-ESR and EULAR-CRP. Differently, when imposing the analysis of the three classes of patients (good, moderate, and non-responders) to the model, only OPLS-DA results according to EULAR-ESR criteria permitted the discrimination between the two groups of responders on LV2 with a good predictivity (Q^2^ = 0.68) and 100% sensitivity and specificity (Fig 1D and S1 Table).

OPLS-DA results according to EULAR-CRP criteria also showed a differentiation between good and moderate responders on LV2, albeit to a lesser extent (Q^2^ = 0.39; 90.9% and 100% sensitivity for good and moderate responders, respectively; 88.9% specificity) (Fig 1E and S1 Table).

Based on OPLS-DA results according to EULAR-ESR criteria, the ^1^H-NMR serum metabolomic profiles of responder patients exhibited higher levels of N-acetylglycoprotein, methionine, pyroglutamate, glutamine, and glucose, as well as lower levels of lactate, arginine, lysine, acetate, sarcosine, aspartate, choline, and formate with respect to non responders. Furthermore, the metabolomic profile of good responders differed from that of moderate responders mainly in the levels of 3-hydroxybutyrate and 1-methyl-hystidine, which were significantly higher, and in the level of alanine, which was significantly lower (Table 4).

**Table 4:** Changes in metabolites in the three groups of patients classified according to EULAR-ESR criteria (good, moderate, and non-responders) after etanercept therapy, as determined by OPLS-DA VIP analysis.

	Responders *vs* non-responders*	Good *vs* moderate responders*	Good responders* after six months of etanercept
**Isoleucine**	**-**	**-**	**↑**
**Leucine**	**-**	**-**	**↑**
**Valine**	**-**	**-**	**↑**
**3-Hydroxybutyrate**	**-**	**↑**	**↓**
**Lactate**	**↓**	**-**	**-**
**Alanine**	**-**	**↓**	**-**
**Arginine**	**↓**	**-**	**-**
**Lysine**	**↓**	**-**	**-**
**Acetate**	**↓**	**-**	**-**
**N-acetylglycoprotein**	**↑**	**-**	**-**
**Glutamine**	**↑**	**-**	**↑**
**Methionine**	**↑**	**-**	**-**
**Pyroglutamate**	**↑**	**-**	**-**
**Sarcosine**	**↓**	**-**	**-**
**Aspartate**	**↓**	**-**	**-**
**Choline**	**↓**	**-**	**-**
**Glucose**	**↑**	**-**	**↑**
**1-Methyl-hystidine**	**-**	**↑**	**-**
**Tyrosine**	**-**	**-**	**↑**
**Formate**	**↓**	**-**	**-**

↑: increased serum levels; ↓ decreased serum levels; -: no change in serum levels.

* EULAR-ESR criteria: EUropean League Against Rheumatism criteria based on erythrocyte sedimentation rate.

### Metabolic Response to Etanercept in Good Responders According to EULAR-ESR Criteria

**Fig 2:**
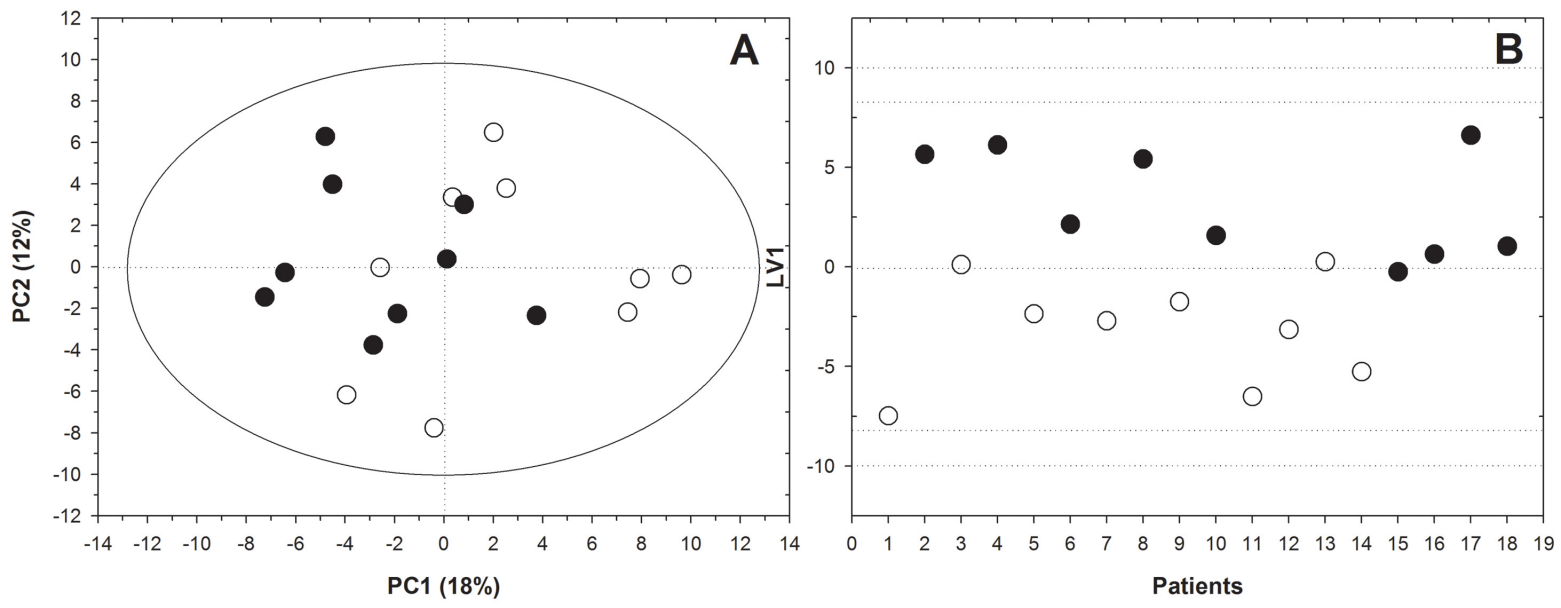
Etanercept caused changes in the metabolism of good responder patients according to EULAR-ESR criteria. Both unsupervised (A) (PCA) and supervised analysis (B)(OPLS-DA) revealed a good separation between serum spectra of patients with a good response to etanercept therapy collected at baseline (white circles) and after six months of treatment (black circles).

In order to further investigate the effect of etanercept treatment on the metabolomic profile of good responders according to EULAR-ESR criteria, we applied a PCA to the dataset of serum samples collected at baseline and after six months of treatment. This approach produced a solution with two significant components, altogether explaining about 30% of the total variability of the systems. A *t*-test was applied to these component scores in order to compare data at baseline and after six months of treatment, which showed significant differences on PC1 (p = 0.021) (Fig 2A). Subsequently, OPLS-DA was used to identify the key discriminatory metabolites. The model separated the metabolic profiles collected at the beginning of observation from those at the end of the experiment with 88.9% sensitivity and 77.7% specificity using one LV (Fig 2B). Analyzing the correlation between each variable and the first LV, we observed increased levels of isoleucine, leucine, valine, glutamine, tyrosine, and glucose and a decreased level of 3-hydroxybutyrate after treatment.

## Discussion

The main aim of this study was to evaluate the possibility of using serum metabolomic profiling as a promising tool for the optimization of biological therapy in the management of RA. This is a critical issue since these drugs fail to produce a response in a substantial proportion of patients. Hence, the optimal treatment strategy should rely on a personalised approach to minimise periods of disease activity and patient exposure to the potential side effects of an ineffective and, in the case of biologics, also expensive treatment. For these reasons, biomarkers of drug response are currently strongly desirable. Recently, Sellam and coll [45] identified a molecular signature that could be predictive of the clinical response to rituximab, as determined by transcriptomic analysis of PBMCs from a large sample of patients with RA. Other non invasive tools might be used to accurately predict the response to biologic drugs in patients with RA.

In this study, we tested the metabolomic profile in a cohort of RA female patients treated with etanercept, classified as responders (good and moderate) or non-responders according to EULAR criteria after six months of therapy. Interestingly, our results showed significant differences in the metabolites found in baseline serum samples of patients who did or did not respond to biological treatment. In particular, by using PCA, an unsupervised analysis, it was possible to discriminate responders and non-responders. When using the OPLS-DA model, a supervised analysis, even a better separation was obtained, demonstrated by an excellent prediction of response to etanercept therapy with 100% sensitivity and specificity. Moreover, the OPLS-DA model based on EULAR-ESR criteria achieved a better discrimination between good and moderate responders with respect to the same model based on EULAR-CRP criteria, whereas the unsupervised analysis PCA failed to find this difference. It is well known that DAS28-ESR and DAS28-CRP scores, used to define EULAR responses, are not interchangeable [46]. Indeed, DAS28-CRP may significantly underestimate disease activity and overestimate the EULAR response criteria compared with DAS28-ESR [46]. Furthermore, the differences in the mean values between the DAS28-ESR and DAS28-CRP may also be affected by patient’s gender, yielding larger scores in females than in males by DAS28-ESR [46,47]. These observations may explain the findings in our cohort of solely female patients about the capability of EULAR-ESR over EULAR-CRP criteria to better discriminate good and moderate responders. Our results are consistent with those reported by Kapoor et al. in urine samples from patients with RA and psoriatic arthritis treated with anti-TNF agents. In this study, ^1^H-NMR spectroscopy revealed a frank relationship between urine metabolic profiles of RA patients at baseline and their response to anti-TNF therapy at twelve weeks. Several metabolites contributed to this difference, in particular histamine, xanthurenic acid, ethanolamine, and glutamine, and this last metabolite was also increased in the serum of responders from our study. Interestingly, we observed decreased levels of lactate in good responders with respect to non-responders, a finding consistent with the observation that elevated concentrations of plasma lactate are widely associated to inflammation [27], and with previous studies supporting lactate as a candidate biomarker for RA severity [20].

Among the disease-related factors that may affect the response to treatment, only the RF-negative status was associated to the response to etanercept (see PC1 in Table 2). This finding is in agreement with the results from a large cohort of RA patients treated with anti-TNF drugs, in whom the presence of RF or ACPA was associated with a reduced response to treatment, although these antibodies only accounted for a small proportion of the variance in the response and the majority was probably explained by genetic factors [48].

To understand whether the level of inflammation might have influenced the response to etanercept, we explored the relationship between the metabolic profiles at baseline as summarized by PCA and the parameters included in the EULAR-CRP and -ESR responses. By using the ANOVA test, we observed a relationship between CRP and PC3 as well as between tender joint count and PC1. However, since the principal components are orthogonal to each other, we can derive that PC3 is independent from PC1, which was able to predict the response to treatment. Therefore, the different metabolic fingerprints of RA patients depending on the level of inflammation did not appear to be predictive of the response to etanercept. Accordingly, although Kapoor et al. observed a significant difference between CRP levels in patients who did or did not respond to TNF antagonists, the partial least-squares regression analysis indicated that the association between urine baseline metabolites and the response was independent of CRP levels [26]. However, this topic is still controversial, as the capability of metabolomics to measure the inflammatory status of RA has been demonstrated in a recent study, in which patients with early arthritis were stratified by ^1^H-NMR metabolomics according to the levels of serum CRP [27].

Finally, metabolomic analysis was used to identify changes in biomarkers after etanercept treatment. Isoleucine, leucine, valine, alanine, glutamine, tyrosine, and glucose levels were found to be increased in good responders as defined by EULAR-ESR criteria, whereas 3-hydroxybutyrate levels were reduced after treatment. Interestingly, Young et al. observed elevated amounts of 3-hydroxybutyrate in RA patients, probably due to the increased lipolysis in the swollen joints of RA patients [49–51]. Therefore, our finding of a decreased concentration of this metabolite in RA patients supports the efficacy of etanercept treatment.

In conclusion, our study confirms the potential of metabolomic analysis to predict the response to biologics. In spite of the limited number of patients, we were able to reproduce our data by using both unsupervised and supervised statistical analyses and obtaining a good natural clustering of metabolomic profiles irrespective of the model applied. Moreover, with regards to responders, this approach allowed to define the metabolic path of the patients along the course of treatment, which may provide further insights on the mechanism of action of biological agents. The future challenges for treating inflammation are clearly the targets to remission. Whether or not remission might be predicted by metabolomic analysis is still unclear and it will be the matter of a future study on a wider cohort of patients.

## Supporting Information

S1 Table Prediction results obtained for OPLS-DA models built for responders *vs* non-responders as well as for good and moderate responders *vs* non-responders according to EULAR-ESR and EULAR-CRP criteria. (DOCX)
