# Supplementary material for: 1H-NMR-Based Metabolomic Study for Identifying Serum Profiles Associated with the Response to Etanercept in Patients with Rheumatoid Arthritis
Source: PLoS One. 2015 Nov 11;10(11):e0138537. doi: 10.1371/journal.pone.0138537 (PMC4641599; doi:10.1371/journal.pone.0138537)
Supplement: S1 Table — (DOCX) [file pone.0138537.s001.docx]

| **MODELS** | **R^2^*** | **Q^2^**** | **Sensitivity** | **Specificity** | **Accuracy** | ***F* probability** |
| --- | --- | --- | --- | --- | --- | --- |
| Responders *vs*  non-responders | 45 | 0.82 | 100 | 100 | 100 | 2.1E-007 |
| Good *vs* moderate responders *vs*  non-responders according to EULAR-ESR criteria | 64 | 0.68 | 100 | 100 | 100 | 2.6E-011 |
| Good *vs* moderate responders *vs*  non-responders according to EULAR-CRP criteria | 23 | 0.39 | 90.9 (good responders)  100 (moderate responders) | 88.9 | 92.59 | 1.3E-009 |

***R^2^:** goodness of fit parameter

****Q^2^:** predictive ability parameter, estimated by cross-validationEULAR-ESR criteria: EUropean League Against Rheumatism criteria based on erythrocyte sedimentation rate; EULAR-CRP criteria: EUropean League Against Rheumatism criteria based on C-reactive protein.

**Supplementary Table 1.** Prediction results obtained for OPLS-DA models built for responders *vs* non-responders as well as for good and moderate responders *vs* non-responders according to EULAR-ESR and EULAR-CRP criteria.
